# Diving into hot topics of salivary gland carcinoma management—an EORTC young and early career investigator survey

**DOI:** 10.3389/fonc.2024.1416097

**Published:** 2024-11-08

**Authors:** Luigi Lorini, Sara Ronchi, Daan Nevens, Konrad Klinghammer, Ester Orlandi, Paolo Bossi, Petr Szturz

**Affiliations:** ^1^ Medical Oncology and Hematology Unit, Scientific Institute for Research, Hospitalization and Healthcare (IRCCS) Humanitas Research Hospital, Milan, Italy; ^2^ Radiation Oncology Unit, Clinical Department, National Center for Oncological Hadrontherapy (CNAO), Pavia, Italy; ^3^ Iridium Netwerk, Radiation Oncology Department, University of Antwerp, Antwerp, Belgium; ^4^ Department of Hematology, Oncology and Cancer Immunology, Charité-Universitätsmedizin, Berlin, Germany; ^5^ Department of Clinical, Surgical, Diagnostic, and Pediatric Sciences, University of Pavia, Pavia, Italy; ^6^ Medical Oncology and Hematology Unit, Department of Biomedical Sciences, Humanitas University, Milan Italy; ^7^ Department of Oncology, University of Lausanne and Lausanne University Hospital, Lausanne, Switzerland

**Keywords:** head and neck, salivary gland, rare tumor, targeted therapy, radiotherapy, surgery

## Abstract

**Introduction:**

Recently, the ASCO and ESMO guidelines on salivary gland carcinomas (SGCs) have been released. However, several crucial points lack strong recommendations due to low or intermediate quality of evidence. To better address these “grey zones” in the guidelines, we conducted a survey among the European Organization for Research and Treatment of Cancer (EORTC) Head and Neck Cancer Group (HNCG) members on behalf of the EORTC young and early career investigators.

**Materials and methods:**

The survey included 29 questions addressing diagnostic and therapeutic issues related to SGC patients and was shared among 539 members of the EORTC HNCG. Responses were collected from December 2022 to March 2023. The primary aim was to evaluate the decision-making criteria guiding physicians in areas with low evidence in SGC guidelines.

**Results:**

With a response rate of 19%, the survey received input from 102 respondents, mainly medical oncologists (45%). Among those with pathological high-risk features, 35% and 30% of respondents opted for chemoradiotherapy (CRT) in the definitive and adjuvant settings, respectively. For patients with R0 resection of highly aggressive SGC at the pT1–2 stage, 37% proposed a close follow-up, while 38% proposed adjuvant lymph-node field RT. In cases of pT3–4 stage, 48% proposed adjuvant lymph-node field RT in all cases, and 44% proposed it based only on risk factors. The most important factors guiding the decision to give adjuvant RT after salvage surgery for locoregional relapse include previous radiotherapy, margin status, and the presence of extranodal extension. When faced with combined positivity to HER2 and AR, responses regarding the choice of first-line palliative treatment were heterogeneous.

**Conclusions:**

International guidelines lack strong recommendations in several fields of SGC diagnosis and treatment due to insufficient high-quality data, resulting in heterogeneity in physicians’ treatment decision (e.g., adjuvant lymph-node field RT and their low concordance with guidelines, such as the use of concomitant CRT). The survey demonstrated the need for prospective clinical trial data to address these gaps in the future.

## Introduction

Major salivary carcinomas (SGCs) are rare tumors, accounting for up to 5% of all newly diagnosed head and neck cancers (HNC), with a reported incidence of 1.3 cases per 100,000 (1). SGCs represent a heterogeneous group of entities with different histology, biology, clinical behavior, and available therapeutic approaches. According to the World Health Organization, SGCs may be divided into low-aggression and high-aggression types (**Table 1**). Acinic cell carcinoma, secretory carcinoma, low-grade or intermediate-grade mucoepidermoid carcinoma, low-grade adenocarcinoma, and cribriform or classic polymorphous adenocarcinoma are classified as low-aggression types, while adenoid cystic carcinoma, poorly differentiated carcinoma, high-grade mucoepidermoid carcinoma, high-grade not otherwise specified (NOS) adenocarcinoma, and high-grade polymorphous adenocarcinoma are considered high-aggression types (2).

**Table 1:** Classification of SGC based on the WHO classification (2022 update).

Low aggression	High aggression
Acinic cell carcinoma	Adenoid cystic carcinoma Tubular/cribriform pattern predominant Solid pattern > 30%
Mammary analog secretory carcinoma	Poorly differentiated carcinoma Neuroendocrine and non-neuroendocrine Undifferentiated carcinoma Large-cell neuroendocrine carcinoma Small-cell neuroendocrine carcinoma
Mucoepidermoid carcinoma Low grade Intermediate grade	Mucoepidermoid carcinoma High grade
Polymorphous Adenocarcinoma Classic Cribriform	Polymorphous High grade
Epithelial–myoephitelial carcinoma	
Hyalinizing clear-cell carcinoma	
Basal cell adenocarcinoma	
Sebaceous adenocarcinoma	Lymphoepithelial carcinoma
Intraductal carcinoma Low grade High grade	Conventional salivary duct carcinoma
Adenocarcinoma not otherwise specified (NOS) Low grade	Adenocarcinoma NOS High grade
Myoepithelial carcinoma	
Oncocytic carcinoma	Carcinosarcoma
Carcinoma ex pleomorphic adenoma risk is determined by the type of carcinoma and extent of invasion.	

The baseline workup for SGCs includes computed tomography (CT) and/or magnetic resonance imaging (MRI), positron emission tomography (PET) with fluorodeoxyglucose, and, in selected cases, PET with prostate-specific membrane antigen (PSMA), along with histological confirmation. In cases of locally/advanced disease, the cornerstone of treatment is surgery, if feasible, followed by radiotherapy. The decision to administer radiotherapy is based on clinical/histological factors, including adenoid cystic histology, high-grade tumors, positive margins, perineural invasion, lymphovascular invasion, pT3–4 tumors, and nodal disease (2–9).

In cases of relapsed and/or metastatic disease (RM) that are no longer amenable to curative treatment, systemic treatment may be offered. Given the rarity and high variability of the disease, managing SGCs is challenging, starting from diagnostic issues to the treatment of localized and advanced diseases. Despite the sensible updates provided by the latest versions of American Society of Clinical Oncology (ASCO) (2) and European Society for Medical Oncology (ESMO) guidelines (3), released in 2021 and 2022, respectively, several crucial points of SGC management remain in a “grey zone”. Due to the scarcity of evidence in the literature, the overall quality of evidence is low, and the strength of recommendations is weak. To complicate matters further, new prognostic and predictive biomarkers have recently opened new treatment opportunities for patients. Molecular alterations such as HER2 amplification, androgen receptor (AR) amplification, and neurotrophic tyrosine receptor kinase (NTRK) fusion are targetable with specific drugs. However, it is unclear how and when to use such drugs, whether in the early phase of the disease within a curative setting following surgery plus radiotherapy (10, 11) or exclusively in the palliative setting (12). Other targetable alterations may be found in SGCs, such as fibroblast growth factor receptor 1 (FGFR1) amplification, activating mutations of the p110α subunit of PI3K (PIK3CA), mutations in the HRAS (mHRAS) proto-oncogene, BRAF mutations, and RET fusion mutation. However, the application of specific drugs targeting these alteration has not been widely available so far (13, 14). Moreover, different molecular alterations in SGCs may be present simultaneously in the same patients. For instance, 30% of AR-positive SGCs have concomitant HER2 amplification (15), 50% have a concomitant PIK3CA mutation, and 41% have a concomitant HRAS mutation (16). Due to the rarity and high biological heterogeneity of the disease, there is a lack of high-quality evidence in the literature, resulting in heterogeneous clinical decision-making approaches among physicians.

We conducted a survey among members of the European Organization for Research and Treatment of Cancer (EORTC) in HNC to explore physicians’ decision-making orientation within the “grey zones” of international guidelines. The questions are divided into main topics: diagnosis (e.g., role and timing of molecular characterization); primary definitive treatment; adjuvant treatment; re-RT after salvage surgery; and palliative treatment (the full questionnaire is presented in the **Supplementary Materials**, **Supplementary Table S1**).

## Materials and methods

The questionnaire was posted as a web survey link on the EORTC HNC mailing list, reaching 539 EORTC members from December 2022 to March 2023. Data were collected via Survey Monkey (www.surveymonkey.com), and descriptive analyses were conducted.

The survey consisted of 29 questions, addressing specific issues in SGC management, covering topics from diagnosis to the treatment of primary disease with curative intent, as well as the treatment of RM disease. The full questionnaire is available in the **Supplementary Materials** (**Supplementary Table S1**).

Based on the ASCO guidelines released in 2021 and the ESMO guidelines released in 2022 regarding the management of SGCs (2, 3), we selected survey questions in accordance with recommendations that are characterized by insufficient to low-intermediate quality of evidence and/or weak to moderate strength of recommendation.

Questions 1 to 8 pertain to the demographic and general data of responders. Questions 9 to 20 were related to treatment with curative intent. Questions 21 to 26 concerned treatment in the RM setting for patients not eligible for curative treatment. Questions 27 to 29 asked participants about their preferred future topics for addressing SGCs. Hereafter, we present the results of our survey.

In order to identify specific “grey zone” topics, we defined “low agreement” questions as those in which 30% or more of respondents disagreed with the guidelines, and “high heterogeneity” questions as those where no single alternative received 50% or more of respondents’ support in single-choice questions, or where multiple alternatives each received 50% or more support in multiple-choice questions.

## Results

### Collection of questionnaires

We reached 102 responses to the questionnaire, with participants primarily from Europe. The majority of responders were from Italy (19%), Belgium (10%), Spain (8%), the UK (8%), France (7%), Germany (7%), and the Netherlands (7%). In total, 55% of responders were aged between 40 and 55 years old. The participants included medical oncologists (45%), radiation oncologists (28%), head and neck surgeons (22%), and other specialists (5%).

Almost half of the participants (45%) stated that they treated 10–20 SGC patients per year. However, 62% reported seeing fewer than 10 new diagnoses of RM SGC per year. The majority of responders (87%) had over 5 years of experience in treating head and neck cancers. Nearly all participants (88%) affirmed that they discussed all SGC cases, both in curative and RM settings, within a multidisciplinary team. Additionally, 69% had access to a molecular tumor board at their hospital. Descriptive data of the responders are provided in **Table 1**. The complete response report is presented in the **Supplementary Materials**.

### Responses with low agreement with guidelines

#### Systemic treatment concomitant with radiotherapy

We characterized the indication for combining chemotherapy and radiotherapy as showing “low agreement” with existing guidelines. Specifically, in cases with pathological high-risk features, 35% respondents indicated they would opt for chemoradiotherapy (CRT) in the definitive, while 30% would do so in the adjuvant setting.

Questions from our survey covered the topic of the combination of systemic treatment with radiotherapy in the curative setting. We inquired about the scenarios in which physicians proposed adjuvant chemotherapy combined with radiotherapy for patients who had undergone surgery (multiple choices were allowed). Factors that inclined physicians toward the addition of chemotherapy included extranodal extension (36%), nonresectable macroscopic residual disease identified on postoperative MRI (30%), and advanced N stage (28%). Interestingly, 23% of respondents stated that they never proposed chemotherapy, while 37% indicated that they would propose chemoradiotherapy only within the context of a clinical trial (**Table 2**).

**Table 2:** Selected questions and answers reported in the main manuscript.

Question	Answer	%
Systemic treatment concomitant with radiotherapy		
In patients treated with adjuvant radiotherapy, when do you combine radiotherapy with chemotherapy? (multiple choices allowed)	a) Based on primary tumor site	a) 1%
	b) Based on tumor histology	b) 24%
	c) In cases of advanced N stage (N2–N3)	c) 28%
	d) In cases of advanced T stage (T3–T4)	d) 16%
	e) Based on age (less than 65 years old)	e) 5%
	f) Based on performance status (ECOG 0–1)	f) 15%
	g) In cases of extranodal extension	g) 36%
	h) In cases of perineural invasion	h) 13%
	i) In cases of lymphovascular invasion	i) 11%
	j) In cases of nonresectable macroscopic residual disease at postoperative magnetic resonance imaging	j) 30%
	k) Only in a clinical trial	k) 37%
	l) Never	l) 23%
In cases of inoperable (due to extension of disease or comorbidity) locally or locoregionally advanced SGC, which treatment options do you propose? (multiple choices allowed)	a) Radiotherapy with photon therapy	a) 38%
	b) Radiotherapy with particle therapy	b) 36%
	c) Chemoradiotherapy	c) 35%
	d) Radiotherapy + targeted anti-HER2 treatment (if HER2 positive)	d) 28%
	e) Radiotherapy + targeted anti-AR treatment (if AR positive)	e) 27%
Radiotherapy indications		
In patients treated with adjuvant radiotherapy, when do you prefer particle therapy (proton or carbon ions) over photons?	a) Based on primary tumor site	*a) *15%
	b) Based on tumor histology	b) 13%
	c) Only in a clinical trial	c) 16%
	d) I cannot offer particle therapy due to logistic issues	d) 21%
	e) Based on patient’s age (less than 65 years old) and performance status (ECOG 0–1)	e) 1%
	f) In cases of nonresectable macroscopic residual disease at postoperative MRI	f) 11%
	g) Never	g) 7%
Systemic treatment		
In cases of HER2-positive SCG (either HER2 3+ on immunochemistry or FISH), when do you offer adjuvant targeted treatment (e.g., trastuzumab)? Multiple choices allowed	a) Based on primary tumor site	a) 1%
	b) Based on tumor histology	b) 11%
	c) In cases of advanced N stage (N2–N3)	c) 18%
	d) In the case of the advanced T stage (T3–T4)	d) 18%
	e) Based on age (less than 65 years old)	e) 2%
	f) Based on performance status (ECOG 0-1)	f) 6%
	g) Only in a clinical trial	g) 50%
	h) In case of nonresectable macroscopic residual disease at postoperative magnetic resonance imaging	h) 23%
	i) Never	i) 25%
In cases of androgen receptor (AR)-positive SGC, when do you offer adjuvant targeted treatment (e.g., leuprolide and/or bicalutamide)? Multiple choices allowed	a) Based on primary tumor site	a) 0%
	b) Based on tumor histology	b) 13%
	c) In cases of advanced N stage (N2–N3)	c) 15%
	d) In cases of advanced T stage (T3–T4)	d) 13%
	e) Based on age (less than 65 years old)	e) 4%
	f) Based on performance status (ECOG 0-1)	f) 7%
	g) Only in a clinical trial	g) 51%
	h) In case of non-resectable macroscopic residual disease at postoperative magnetic resonance imaging	h) 28%
	i) Never	i) 22%
In a candidate for systemic treatment with both HER2-positive and AR-positive SGC, which systemic treatment do you prefer in the first line?	a) HER2 targeted treatment (e.g., trastuzumab)	a) 28%
	b) AR targeted treatment (e.g., leuprolide and/or bicalutamide	b) 21%
	c) Combination of HER2 and AR targeted treatment	c) 18%
	d) HER2-targeted treatment + chemotherapy	d) 23%
	e) AR-targeted treatment + chemotherapy	e) 1%
	f) Chemotherapy	f) 8%

Moving to the definitive setting, for patients with locally or locoregionally advanced SGCs who were not candidates for surgery due to disease extension or comorbidities, 35% of responders proposed chemoradiotherapy. Additionally, 38% recommended only radiotherapy with photons, 36% suggested radiotherapy with particle therapy, and 28% advocated radiotherapy combined with targeted treatment if the patient’s receptors were positive (AR or HER2) (**Table 2**).

### Responses with high levels of heterogeneity

#### Radiotherapy indications

In terms of primary treatment with curative intent, we presented two similar cases of high-aggression SGCs that were surgically treated with curative intent, resulting in an R0 status in the histopathological report, with no regional lymph node involvement (cN0). For pT1-2 stage cases, we asked about the treatment approach: 37% suggested close follow-up, 38% recommended adjuvant lymph-node field radiotherapy if needed for the primary site (e.g., in the presence of perineural invasion and/or lymphovascular invasion), 14% proposed elective neck dissection, and 6% suggested adjuvant lymph-node field radiotherapy in every case. For pT3–4 stage cases, we asked whether responders felt confident proposing adjuvant lymph-node field radiotherapy; 48% proposed it in every case, while 44% based their decision on the primary tumor site, histology, and neck dissection.

Following these questions, 74% of responders expressed the need for contouring guidelines to define target volumes for patients treated with radiotherapy for SGCs. Respondents were then asked about cases where they preferred adjuvant radiotherapy using particle therapy over photon therapy. Notably, 21% could not propose particle therapy due to logistical issues. Decisions were primarily based on the primary tumor site (15%), tumor histology (13%), and the presence of nonresectable macroscopic residual disease identified on postoperative MRI (11%). Additionally, 16% preferred particle therapy only within the context of a clinical trial. Specifically for adenoid cystic carcinoma, 26% preferred carbon ion radiotherapy (CIRT), while 21% preferred proton therapy (**Table 2**).

In cases of locoregional relapse (following primary disease treatment based on surgery and radiotherapy) treated with surgery, physicians were asked to rate the importance of several factors in the decision-making process for proposing adjuvant radiotherapy. Factors considered “very important” included R2 resection (79%), previous radiotherapy (timelapse not indicated) (78%), R1 resection (68%), presence of extranodal extension (ENE) (60%), and presence of perineural invasion (40%). Notably, only 36% of responders based their decision on tumor histology (**Table 3**).

**Table 3:** Question 21: In patients with locoregional relapse treated with surgery, please rate the degree of importance of the following factors in your decision-making regarding adjuvant radiotherapy.

Factors to be considered	Very important	Important	Neutral	Not so important	Not important	Total number of responders
	n (%)	n (%)	n (%)	n (%)	n (%)	
*Disease interval*	36 (39.6)	38 (41.8)	13 (14.2)	1 (1.1)	3 (3.3)	91
*Previous radiotherapy*	75 (78.1)	21 (21.9)	0	0	0	96
*Tumor grade*	21 (23.3)	52 (57.8)	12 (13.4)	3 (3.3)	2 (2.2)	90
*Tumor histology*	34 (37.0)	45 (48.8)	10 (10.9)	2 (2.2)	1 (1.1)	92
*R1 resection*	64 (68.1)	23 (24.4)	6 (6.4)	1 (1.1)	0	94
*R2 resection*	76 (79.2)	18 (18.7)	2 (2.1)	0	0	96
*Presence of extranodal extension*	57 (60.0)	33 (34.7)	4 (4.2)	0	1 (1.1)	95
*Presence of perineural invasion*	37 (40.2)	46 (50.0)	7 (7.6)	1 (1.1)	1 (1.1)	92
*Presence of lymphovascular invasion*	23 (25.3)	54 (59.3)	9 (9.9)	4 (4.4)	1 (1.1)	91
*rT stage*	33 (35.1)	51 (54.3)	8 (8.5)	1 (1.1)	1 (1.1)	94
*rN stage*	36 (37.9)	52 (54.7)	5 (5.2)	1 (1.1)	1 (1.1)	95
*Performance status*	36 (39.1)	47 (51.1)	9 (9.8)	0	0	92
*Age*	12 (13.5)	44 (49.4)	25 (28.1)	6 (6.7)	2 (2.3)	89
*Only in a clinical trial*	2 (2.7)	13 (17.6)	34 (46.0)	14 (18.8)	11 (14.9)	74
*I never propose adjuvant radiotherapy for locoregional relapse*	1 (1.6)	1 (1.6)	24 (38.7)	6 (9.7)	30 (48.4)	62

#### Systemic treatment

In the postoperative setting, for HER2-positive SGCs (either HER3+ on immunochemistry or FISH) or AR-positive cases, physicians were asked if they would offer adjuvant targeted treatments (e.g., trastuzumab or leuprolide and/or bicalutamide, respectively). The majority of responders (49% and 50%, respectively) indicated they would offer targeted treatment if feasible within the context of a clinical trial (**Table 2**).

In the palliative setting, the impact of various characteristics influencing physicians’ decisions to initiate systemic treatment over close follow-up was evaluated. Factors considered “very important” included the presence of symptoms (69%), burden of disease (54%), tumor histology (53%), patient preferences (50%), and patient performance status (45%) (**Table 4**).

**Table 4:** Question 24: Which factors influence your decision to start systemic treatment rather than to closely follow-up patients?

Factors to be considered	Very important	Important	Neutral	Not so important	Not important	Total number of responders
	n (%)	n (%)	n (%)	n (%)	n (%)	
*Tumor histology*	50 (53.8)	31 (33.2)	10 (10.8)	1 (1.1)	1 (1.1)	93
*Disease-free interval between termination of prior therapy and relapse*	32 (33.0)	45 (46.4)	15 (15.4)	3 (3.1)	2 (2.1)	97
*Symptoms*	69 (69.0)	24 (24.0)	7 (7.0)	0	0	100
*Burden of disease*	54 (54.0)	33 (33.0)	9 (9.0)	4 (4.0)	0	100
*Pace of disease*	39 (41.5)	38 (40.4)	12 (12.7)	4 (4.3)	1 (1.1)	94
*Age*	20 (20.4)	46 (47.0)	24 (24.5)	6 (6.1)	2 (2.0)	98
*Gender*	1 (1.1)	6 (6.5)	39 (42.4)	8 (8.7)	38 (41.3)	92
*Presence of liver or bone metastasis*	24 (24.7)	48 (49.5)	21 (21.7)	1 (1.0)	3 (3.1)	97
*Patient preferences*	48 (50.0)	39 (40.6)	7 (7.4)	1 (1.0)	1 (1.0)	96
*Performance status*	45 (45.0)	49 (49.0)	4 (4.0)	2 (2.0)	0	100
*Risk of toxicity*	34 (34.7)	49 (50.0)	13 (13.3)	2 (2.0)	0	98
*Availability of a clinical trial*	44 (44.4)	40 (40.4)	11 (11.1)	1 (1.1)	3 (3.0)	99

Finally, we presented a clinical scenario involving a patient affected by recurrent or metastatic SGC eligible for systemic treatment, exhibiting both HER2 and AR positivity. Responses varied: 27% proposed HER2-targeted treatment, 22% suggested chemotherapy combined with HER2-targeted treatment, 21% opted for androgen receptor-targeted treatment, 18% recommended a combination of anti-HER2 and anti-AR treatments, and 8% suggested chemotherapy alone (**Table 2**).

## Discussion

The purpose of this extensive survey was to investigate the various grey areas that exist in the management of salivary gland cancers, as reported in published guidelines (2, 3), and to gain insights from current clinical practice.

### Systemic treatment concomitant with radiotherapy

The first point that emerged from our survey is the role of systemic treatment concomitant with radiotherapy in both adjuvant and definitive settings. We found low agreement between responders and existing guidelines on this topic. ASCO and ESMO guidelines do not recommend the use of concomitant chemotherapy in adjuvant settings outside of clinical trials (with a moderate strength of recommendation) (2, 3, 17–19). In contrast, NCCN guidelines recommend chemoradiotherapy in selected cases (with a 2B level of recommendation) (20). In our survey, 22% of responders agree with ASCO and ESMO indications, while the remaining, consider chemotherapy with radiotherapy, mainly in cases of highly aggressive disease, advanced N or T stage, presence of extranodal extension, or R1 resection. Notably, one out of three responders consider the use of chemotherapy only within a clinical trial. RTOG 1008 (NCT01220583) is an ongoing phase III trial randomizing patients with SGCs to receive adjuvant radiation with or without concurrent cisplatin. Similarly, the French phase III SANTAL trial (NCT02998385) is investigating the addition of concurrent chemotherapy to curative adjuvant radiation (to note, the study comprises even unresectable SGCs). Given the lack of data on such a topic, based on the results of our survey, the physicians are more inclined to follow “clinical” characteristics of the disease and the patients rather than the histological features, often translating data from squamous cell carcinoma of the head and neck.

In contrast to chemotherapy, data on HER2- and AR-targeted adjuvant treatments following radiotherapy have demonstrated clinical benefits (10, 21). Given these impressive results, 50% of responders in our survey indicated they would use AR- or HER2-targeted treatment as an adjuvant strategy, albeit limited to the context of a clinical trial. This aligns with guidelines that suggest the use of HER2- or AR-directed adjuvant treatment exclusively within clinical trials (2, 3).

Similarly, there are no prospective data comparing the use of chemoradiotherapy with radiotherapy as a definitive treatment for nonrectable disease; thus, guidelines do not recommend chemoradiotherapy as a definitive treatment (with a moderate strength of recommendation) (2, 3, 22). In contrast with such a statement, 36% of responders in our survey indicated they would consider chemoradiotherapy for inoperable locally or locoregionally advanced SGCs. The intent is clearly to achieve higher locoregional control and reduce the risk of distant relapses. However, given the paucity of data, our survey results indicate that evaluations are made on a case-by-case basis, often translating practices from head and neck squamous cell carcinomas, by reserving the addition of chemotherapy for young, symptomatic patients with high-volume tumors and high-aggression histology, particularly those with a Ki67 that may predict responsiveness to chemotherapy, in order to counterbalance the burden of toxicities.

### Radiotherapy indications

The second topic that emerged from our survey is the role of adjuvant RT after surgical resection of SGCs in cases of locoregional relapse, which showed remarkably high heterogeneity among responders. International guidelines (2, 3) strongly recommend postoperative radiotherapy in the primary tumor region, endorsing it as a clear indication for all patients with resected adenoid cystic carcinomas, as well as for those with adverse features such as T3–T4 disease, high/intermediate-grade disease, close or incomplete resection margins, and/or perineural growth. In fact, in such cases, adjuvant RT showed a significant improvement in local control (LC) and overall survival (OS). Postoperative RT also improved regional control from 62% to 86% in patients with pN+ neck involvement (23).

Very recently, in September 2024, the published REFCOR guidelines considered additional factors and recommend postoperative radiotherapy to the primary tumor site if one or more of the following adverse histoprognostic factors are present (risk > 10% of locoregional recurrence): T3–T4 category, lymph node invasion, extraglandular invasion, close or positive surgical margins, high tumor grade, perineural invasion, vascular emboli, and/or bone invasion. For unresectable cancers or inoperable patients, carbon ion hydrotherapy may be considered (24).

Factors deemed very important by the majority of responders included previous radiotherapy (78%), R2 resection (79%), R1 resection (68%), and the presence of extranodal extension (0%). Notably, only 36% of responders considered tumor histology to be “very important”. This is quite surprising and contrasts with the guidelines, which indicate that histology is one of the key factors in deciding whether to administer adjuvant radiotherapy to the primary tumor (9). Physicians seem to translate data from the treatment of squamous cell carcinoma of the head and neck (23), where R1 resection and extranodal extension play a significant role in determining adjuvant treatment decisions after curative surgery, and the importance of tumor grading and histology is less clearly defined. One specific histology that often portends an increased risk for local recurrence is adenoid cystic carcinoma. Single-institution, retrospective analyses have suggested that patients with adenoid cystic carcinoma experience improved locoregional control with the addition of adjuvant RT. This is predominantly due to the carcinoma’s propensity for neurotropic spread along cranial nerves (26). The lack of trials addressing this specific issue and the consequent lack of strong recommendations in the guidelines may explain the high heterogeneity among responders. Notably, eight out of 10 responders consider previous radiotherapy to be a limiting factor. However, the timeframe for considering reirradiation is not clear, making it essential to investigate how long it would take for this approach to be deemed safe for patients. In our survey, the results regarding the indication for adjuvant RT in cases of positive or close margins were consistent with the guidelines (80% of responders choose adjuvant RT for all cases of highly aggressive tumors with close margins; in this scenario, factors such as advanced stage and perineural invasion were rated as very important by the majority of responders, followed by lymphovascular invasion, while the site of disease was mostly viewed as an important or neutral factor). The international guidelines recommend postoperative RT for R1 margins following surgery (2, 3, 24). However, the issue of surgical radicalization is not directly addressed. We asked about the most important factors guiding clinicians in their choice of adjuvant RT versus eventual re-resection after R1 surgery. Responders indicated that the possibility of functional impairment (85%) was the most important factor. In fact, re-resection should be considered with caution due to the risks of morbidity, especially when other adverse features, such as high-aggressive histology, advanced stage, or perineural invasion, make the patient eligible for RT.

When managing these rare diseases in clinical practice, especially when patients have not been previously treated by surgery at referral centers, clinicians may encounter “gray zones” regarding the indications for adjuvant RT. The decision on when to administer RT to the surgical bed and/or when to perform elective nodal irradiation (ENI) can vary significantly among clinicians.

The third topic that emerged from our survey is the role of adjuvant treatment in highly aggression SGCs resected with negative margins but without neck dissection performed and a clinically negative neck (cN0), as evaluated through a hypothetical clinical scenario. For pT1–2 stages, the majority of responders proposed either close follow-up (37%) or adjuvant lymph-node field radiotherapy if radiotherapy was required for the primary site (38%) (e.g., if the presence of perineural invasion and/or lymphovascular invasion). Only 14% proposed elective neck dissection, and 6% proposed adjuvant lymph-node field radiotherapy in every case. Surprisingly, the majority of responders were nearly evenly divided between two very different clinical approaches: a more conservative strategy (close follow-up) and a more aggressive approach (adjuvant lymph-node RT if there was a coexisting indication for RT at the primary site). Despite international guidelines recommending postoperative RT in all cases of adenoid cystic carcinoma (ACC), based on single-institution data (26), and in the presence of adverse features (such as high grade, perineural invasion, or lymphovascular invasion), responders’ perception of the importance of highly aggressive histology in guiding adjuvant RT still appears quite variable. A not-negligible number of clinicians (37%) give more importance to early stage and negative margins over highly aggressive histology in their decision-making. Once again, some clinicians seem to translate data from squamous cell carcinoma of the head and neck treatment (25). On the contrary, a significant proportion of responders assigned high importance to highly aggressive histology regardless of stage or R0 considering adding lymph-node volumes whenever postoperative RT was indicated. The high heterogeneity in responses may reflect the difficulty of framing the decision-making process within categorized approaches, as well as the uncertainty caused by the rarity of SGCs, their variegated spectrum of histological types, different biological behaviors, and changes in histological classifications over time.

The fourth point that emerged from our survey is whether the RT field should include only the surgical bed, given that the strength of recommendation for elective neck irradiation in patients with cN0 disease, T3–T4 cancers, or high-grade malignancies is considered moderate.

We proposed a clinical case of high-aggression SGCs following radical resection with a clinically negative neck. In the case of the pT3–4 stage, we asked whether responders felt confident proposing adjuvant lymph-node field radiotherapy. Forty-eight percent proposed an adjuvant lymph-node field radiotherapy in every case, while 44% based their decision on factors such as primary tumor site, histology, and whether neck dissection had been performed. Following these questions, 74% of responders expressed the need for contouring guidelines to define target volumes in patients treated with RT for SGCs, following the example of existing contouring guidelines for perineural spread (27).

### Systemic treatment

The fifth point that emerged from our survey is the lack of strong recommendations for systemic treatment in cases of RM disease that are not amenable to curative treatment.

Palliative systemic management of RM SGC has been traditionally relied on chemotherapy, typically reserved for symptomatic patients with a high disease burden and rapid growth. Recently, the identification of targetable molecular alterations has expanded treatment opportunities. However, the complexity increases as the same disease may carry different molecular alterations, and a correct algorithm of treatment for such cases has yet to be developed. The absence of international indication in these situations is evident in the heterogeneity of responses to a clinical scenario involving a patient with concomitant HER2 amplification and AR expression. Twenty-seven percent of respondents would choose HER2-targeted treatment, 22% for a combination of HER2-targeted treatment and chemotherapy, 21% for AR-targeted treatment, and 18% for a combination of HER2- and AR-targeted treatment. Discussion within a molecular tumor board, available at 69% of respondents’ centers, is essential in such cases, highlighting the importance of centralizing the treatment of such rare tumors. Moreover, lessons may be learned from other histological types. For instance, in breast cancer (BC), the coexpression of HER2 and AR amplification has an unclear impact on prognosis, which differs from the context of SGCs. Preclinical data on HER2-positive BC showed a reduction in tumor growth with enzalutamide, both as a single agent and in combination with T. However, clinical applications of this approach are lacking (28).

In adjunct to the obvious limitations of such a survey in general, it is important to consider that the current survey was released in December 2022, while the ASCO and ESMO guidelines were published in June 2021 and November 2022, respectively (2, 3). Consequently, responders may have been more influenced by the ASCO guidelines than the ESMO guidelines, despite the fact that the two guidelines do not show substantial differences. Another limitation is the low proportion (28%) of radiation oncologists among responders, which may lead to a limited perspective on radiation treatment topics that have a pivotal role in SGC treatment. Moreover, the clinical scenario proposed may have missed some essential clinical information; however, it was proposed following indications/sentences posed by the guidelines.

The primary aim of the current survey was to explore the grey zones of the guidelines, ultimately identifying opportunities for prospective trials to address unmet medical needs. Ninety percent of respondents expressed interest in participating in future clinical trials on these topics. Based on the results of our survey, the main concerns can be divided into issues related to radiotherapy and systemic treatment.

From a radiotherapy point of view, further data are needed to better define the volume and factors to consider for the nodal coverage after surgery and the expanding role of particle therapy treatment.

From a systemic treatment point of view, molecular characterization offers new opportunities; however, some points need to be better explored. Which method should be preferred for molecular analysis: immunohistochemical, next-generation sequencing, or gene expression analysis?

Moreover, is it possible to implement targeted treatment in the curative setting, following the examples of AR- and HER2-targeted treatment in the palliative setting? How concomitant mutations should be managed? These questions are still without clear answers, highlighting the need for multicenter international prospective clinical trials to better explore prognostic and predictive biomarkers and to develop a tailored treatment for SGC patients. Recently, at ESMO 2024, intriguing data have been presented on this topic. Trastuzumab deruxtecan appears promising for HER2-positive RM SGCs, although definitive data and data on HER2-low SGCs are still pending (29). Furthermore, even if ADT compared to standard of care did not show improved PFS in AR-positive SGCs, the authors concluded that ADT combined with chemotherapy and/or with HER2 inhibitors, depending on HER2 status, may represent a rational approach for future studies (30).

Additional comments on the survey results are provided in the **Supplementary Material**.

## Conclusion

The “grey zones” of ASCO and ESMO guidelines reflect a high heterogeneity and low concordance with physician’s choices in the management of SGCs. Further clinical trials are needed to generate higher-quality data in order to support physicians’ decisions and to achieve more homogeneity in the treatment of this rare and challenging disease.

## Data Availability

The original contributions presented in the study are included in the article/**Supplementary Material**. Further inquiries can be directed to the corresponding author.
