# Supplementary material for: Diving into hot topics of salivary gland carcinoma management—an EORTC young and early career investigator survey
Source: Front Oncol. 2024 Nov 8;14:1416097. doi: 10.3389/fonc.2024.1416097 (PMC11582063; doi:10.3389/fonc.2024.1416097)
Supplement: Supplementary file 1 [file DataSheet1.docx]

**Additional Comments**

*- Adjuvant Systemic Treatment*

Retrospective series did not show an advantage in overall survival (OS) and progression free survival (PFS) in giving chemoradiotherapy instead of radiotherapy alone in surgical resected salivary gland carcinoma (SGC). It is to consider that chemotherapy has been used in patients with late stage and adverse features, with N+, perineural invasion (PNI), and R1 resection (1–3).

Differently from chemotherapy, data of HER2 and androgen receptor (AR) targeted adjuvant treatment, after radiotherapy, showed clinical benefit. A retrospective series on 22 patients with high risk AR positive salivary ductal carcinoma receiving bicalutamide or LHRH analog showed a median disease free survival (DFS) of 33 months, compared with a control group with DFS of 21 months (4). Increase in DFS (117 vs 9 months) if compared with control group have been observed even for HER2-positive or neu-positive (IHC 3+) salivary ductal carcinoma (SDC) receiving adjuvant trastuzumab after radiotherapy (RT) (5). An ongoing trial is evaluating TDM-1 in adjuvant setting in HER2 positive SGC (NCT04620187). Given the impressive results, 50% of responders of our survey would use AR or HER targeted treatment as adjuvant strategy, up to now limited in the context of a clinical trial, indication in accord with guidelines that suggest the use of HER or AR directed adjuvant treatment only within a clinical trial. However, the clinical benefit for patients, with a relative low burden of toxicities, made such approach attractive, and data from prospective trials are urgently needed for the wide introduction of such treatment in the curative scenario, also in order to reduce the use of chemotherapy that seems not to guarantee adequate clinical benefit if compared with the burden of toxicities. With a window on the future, given the increase in findings of predictive biomarkers (6), and by translating data from other solid tumors, such as gastrointestinal stromal tumor and non-small cell lung cancer (7–9) it will be of interest to evaluate even other molecular targets in the curative setting, and not to reserve them just for the palliative setting.

*- Adjuvant treatment after surgical treatment of locoregional relapse*

Referring on literature using matched pair analysis or large retrospective cohort studies, international guidelines recommend postoperative radiotherapy at the primary tumor region endorsing it as a clear indication for all patients with resected adenoid cystic carcinomas and in case of adverse features such as T3-T4 disease, high/intermediate-grade disease, close or incomplete resection margins and/or perineural growth. In fact, in such cases, adjuvant RT showed significant improvement in local control (LC) and overall survival (OS) (1,10–13). Post-operative RT also resulted in improved regional control from 62% to 86% in patients with neck node involvement at pathology.

*- Role of particle therapy*

Concerning the radiotherapy technique, we asked if particle therapy could be considered as an option instead of photons. Salivary gland cancers are radioresistant tumors requiring high radiation doses, often in critical sites (e.g. in case of perineural invasion, skull base invasion, nasal/paranasal location), often representing a challenge for radiation oncologists. In this scenario, thanks to the peculiar physical and radiobiological characteristics, particle therapy may be an option as indicated both by ASCO (14) and ESMO guidelines (15). In particular, adenoid cystic carcinoma is a good candidate for carbon ion radiotherapy based on a radiobiological rationale (16) and clinical experiences. In a curative setting, photon radiotherapy showed in retrospective series (including predominantly T4 stage-disease) 5-year locoregional control rates of up to 50% with 10-year rates reaching only 30% (12,13,17). In similar advanced-stage patient series, primary particle therapy, namely carbon ions, showed better results with 5-year local control rates of 60%-70% (18,19). However, there are no prospective studies directly comparing photons with protons and no randomized trials comparing photons with carbon ions.

When we asked in our survey about particle therapy in a curative setting (inoperable locally advanced disease or R2 after surgery), the majority of responders (39%) declared to prefer particle therapy over photon radiotherapy (23%) reflecting confidence in the prospective evidence showing higher control rates. However, the amount of responders choosing concurrent chemoradiation was almost comparable (34%), underlying that the low evidence level caused by the lack of results from randomized trials for both approaches, lead to heterogeneity in clinical practice, with choice probably influenced also by different availability/accessibility of particle therapy facilities. These issues reflect also in the adjuvant setting (21% of responders not offering particle radiotherapy due to logistic issues, and 16% considering it only within a clinical trial) and when specifically addressing to adenoid cystic carcinoma, where responders are almost equally divided between those not proposing particle therapy (52%) and those proposing it (48%). In particular, among the latter, more prefer carbon ions (27%) than protons (21%), despite the reduced number of carbon ion facilities across Europe when compared to proton ones (20). Maybe this could be explained by higher confidence in the carbon ion experience of the last decades (prospective studies, higher number of patients in the literature series). Our survey confirm the field of particle therapy in SCG treatment as a “grey zone” also in clinical practice reflecting in a high heterogeneity of treatment approaches. It is not clear to what extent this heterogeneity is due to the limited availability of particle facilities or to the low level of evidence. This stresses the need, from one hand, to promote collaboration between centers also in an international landscape optimizing patients’ referral to particle therapy facilities, from the other hand, to build up multicentric prospective and possibly randomized trials to better clarify the role of particle therapy.

*- Systemic Palliative Treatment*

Moving to palliative systemic management of recurrent/metastatic (RM) SGC, treatment of SGC have been traditionally based on chemotherapy, that up to now, has usually been reserved to symptomatic patients with a high growth rate and high burden of disease. Recently, targetable molecular alterations have increased the treatment opportunities, especially in non-ACC tumors, such as androgen receptor expression (up to 98% of SDC), HER2 amplification (both in SDC about 20-30% of cases, and adenocarcinoma about 20% of cases), neurotrophic tyrosine receptor kinase (NTRK) gene fusions in secretory carcinoma (about 80% of cases), or rarer as Fibroblast growth factor receptor 1 (FGFR1) amplification, activating mutations of the p110α subunit of PI3K (PIK3CA), mutations in the HRAS (mHRAS) proto-oncogene, BRAF mutation, and RET fusions (21). However, despite improved knowledge on tumor biology and expanding therapeutic options, the correct timing to start systemic treatment over close follow up is still shrouded in the fog. In our survey, factors rated as very important to consider for systemic treatment were presence of symptoms (69%), high burden of disease (54%), tumor histology (53%), patients´ preferences (50%), patients’ performance status (45%), and pace of disease (41%). Such indications are in line with guidelines (14,15), however, they seem to be related with an indication to chemotherapy rather than targeted treatment. Of note, as recommended by guidelines (14,15), 44% of respondents prefer to refer patients to clinical trial in the first instance, if available.

*- Palliative treatment with concomitant molecular alterations*

Fifty % and 41% of AR positive SDC harbored PIK3CA and HRAS mutations respectively (22). A case series reported initial promising data combining AR directed treatment and HRAS or PIK3CA target treatment (22). Combination or sequential therapies for multiple targeted treatments, warrant further investigations, ideally in clinical trials. Discussion in the context of a molecular tumor board, (available in 69% of respondents’ centers) is essential in such cases, highlighting once again the importance to centralize such rare tumors.

REFERENCES

1. Cheraghlou S, Kuo P, Mehra S, Agogo GO, Bhatia A, Husain ZA, et al. Adjuvant therapy in major salivary gland cancers: Analysis of 8580 patients in the National Cancer Database. Head Neck. 2018 Jul;40(7):1343–55.

2. Mifsud M, Sharma S, Leon M, Padhya T, Otto K, Caudell J. Salivary Duct Carcinoma of the Parotid: Outcomes with a Contemporary Multidisciplinary Treatment Approach. Otolaryngol Neck Surg. 2016 Jun;154(6):1041–6.

3. Gebhardt BJ, Ohr JP, Ferris RL, Duvvuri U, Kim S, Johnson JT, et al. Concurrent Chemoradiotherapy in the Adjuvant Treatment of High-risk Primary Salivary Gland Malignancies. Am J Clin Oncol. 2018 Sep;41(9):888–93.

4. Van Boxtel W, Locati LD, Van Engen-van Grunsven ACH, Bergamini C, Jonker MA, Fiets E, et al. Adjuvant androgen deprivation therapy for poor-risk, androgen receptor–positive salivary duct carcinoma. Eur J Cancer. 2019 Mar;110:62–70.

5. Hanna GJ, Bae JE, Lorch JH, Haddad RI, Jo VY, Schoenfeld JD, et al. The Benefits of Adjuvant Trastuzumab for HER-2-Positive Salivary Gland Cancers. The Oncologist. 2020 Jul 1;25(7):598–608.

6. Cleymaet R, Vermassen T, Coopman R, Vermeersch H, De Keukeleire S, Rottey S. The Therapeutic Landscape of Salivary Gland Malignancies—Where Are We Now? Int J Mol Sci. 2022 Nov 28;23(23):14891.

7. Laurent M, Brahmi M, Dufresne A, Meeus P, Karanian M, Ray-Coquard I, et al. Adjuvant therapy with imatinib in gastrointestinal stromal tumors (GISTs)—review and perspectives. Transl Gastroenterol Hepatol. 2019 Apr;4:24–24.

8. Sposito M, Belluomini L, Pontolillo L, Tregnago D, Trestini I, Insolda J, et al. Adjuvant Targeted Therapy in Solid Cancers: Pioneers and New Glories. J Pers Med. 2023 Sep 22;13(10):1427.

9. Tsuboi M, Herbst RS, John T, Kato T, Majem M, Grohé C, et al. Overall Survival with Osimertinib in Resected *EGFR* -Mutated NSCLC. N Engl J Med. 2023 Jul 13;389(2):137–47.

10. Zeidan YH, Shultz DB, Murphy JD, An Y, Chan C, Kaplan MJ, et al. Long‐term outcomes of surgery followed by radiation therapy for minor salivary gland carcinomas. The Laryngoscope. 2013 Nov;123(11):2675–80.

11. Terhaard CHJ, Lubsen H, Van der Tweel I, Hilgers FJM, Eijkenboom WMH, Marres HAM, et al. Salivary gland carcinoma: independent prognostic factors for locoregional control, distant metastases, and overall survival: results of the Dutch head and neck oncology cooperative group. Head Neck. 2004 Aug;26(8):681–93.

12. Terhaard CHJ, Lubsen H, Rasch CRN, Levendag PC, Kaanders HHÀM, Tjho-Heslinga RE, et al. The role of radiotherapy in the treatment of malignant salivary gland tumors. Int J Radiat Oncol. 2005 Jan;61(1):103–11.

13. Chen AM, Bucci MK, Weinberg V, Garcia J, Quivey JM, Schechter NR, et al. Adenoid cystic carcinoma of the head and neck treated by surgery with or without postoperative radiation therapy: Prognostic features of recurrence. Int J Radiat Oncol. 2006 Sep;66(1):152–9.

14. Geiger JL, Ismaila N, Beadle B, Caudell JJ, Chau N, Deschler D, et al. Management of Salivary Gland Malignancy: ASCO Guideline. J Clin Oncol. 2021 Apr 26;JCO.21.00449.

15. van Herpen C, Vander Poorten V, Skalova A, Terhaard C, Maroldi R, van Engen A, et al. Salivary gland cancer: ESMO–European Reference Network on Rare Adult Solid Cancers (EURACAN) Clinical Practice Guideline for diagnosis, treatment and follow-up. ESMO Open. 2022 Nov;100602.

16. Loap P, Vischioni B, Bonora M, Ingargiola R, Ronchi S, Vitolo V, et al. Biological Rationale and Clinical Evidence of Carbon Ion Radiation Therapy for Adenoid Cystic Carcinoma: A Narrative Review. Front Oncol. 2021 Nov 30;11:789079.

17. Holtzman A, Morris CG, Amdur RJ, Dziegielewski PT, Boyce B, Mendenhall WM. Outcomes after primary or adjuvant radiotherapy for salivary gland carcinoma. Acta Oncol. 2017 Mar 4;56(3):484–9.

18. Sulaiman NS, Demizu Y, Koto M, Saitoh J ichi, Suefuji H, Tsuji H, et al. Multicenter Study of Carbon-Ion Radiation Therapy for Adenoid Cystic Carcinoma of the Head and Neck: Subanalysis of the Japan Carbon-Ion Radiation Oncology Study Group (J-CROS) Study (1402 HN). Int J Radiat Oncol. 2018 Mar;100(3):639–46.

19. Akbaba S, Ahmed D, Mock A, Held T, Bahadir S, Lang K, et al. Treatment Outcome of 227 Patients with Sinonasal Adenoid Cystic Carcinoma (ACC) after Intensity Modulated Radiotherapy and Active Raster-Scanning Carbon Ion Boost: A 10-Year Single-Center Experience. Cancers. 2019 Nov 1;11(11):1705.

20. https://www.ptcog.site/index.php/facilities-in-operation-public.

21. Locati LD, Ferrarotto R, Licitra L, Benazzo M, Preda L, Farina D, et al. Current management and future challenges in salivary glands cancer. Front Oncol. 2023 Sep 19;13:1264287.

22. Rieke DT, Schröder S, Schafhausen P, Blanc E, Zuljan E, Von Der Emde B, et al. Targeted treatment in a case series of AR+, HRAS/PIK3CA co-mutated salivary duct carcinoma. Front Oncol. 2023 Jun 20;13:1107134.

**Table**

| **General part** | | | **Results** |
| --- | --- | --- | --- |
| 1 | Which age category do you belong | 1. < 40 2. 40-55 3. >55 | 1. 25% 2. 55% 3. 20% |
| 2 | Which country do you practice? |  | 1. Italy 20% 2. Belgium 11% 3. Spain 9% 4. United Kingdom 9% 5. France 8% 6. Germany 8% 7. Netherlands 8% 8. Switzerland 5% 9. Other 22% |
| 3 | What is your specialty? | 1. Medical Oncologist 2. Radiation Oncologist 3. Otolaryngology 4. Clinical Oncologist 5. Other (please specify) | 1. 37% 2. 28% 3. 23% 4. 9% 5. 3% |
| 4 | How many years have you experience in treating head and neck cancer patients? | 1. < 5 years 2. 5-15 years 3. > 15 years | 1. 13% 2. 44% 3. 43% |
| 5 | How many new diagnoses of salivary gland cancer (SGC) do you see in your center per year (irrespective of disease stage)? | 1. < 10 2. 10-20 3. > 20 | 1. 21% 2. 45% 3. 34% |
| 6 | How many new cases of Recurrent and/or Metastatic (RM) SCG do you see in your center per year? | 1. < 10 2. 10-20 3. > 20 | 1. 63% 2. 32% 3. 5% |
| 7 | Which cases of SGC are discussed by your multidisciplinary team? | 1. All cases with curative intent 2. All cases with curative intent AND palliative intent 3. Selected cases | 1. 6% 2. 88% 3. 6% |
| 8 | Is a molecular tumor board available at your center? | 1. Yes 2. No | 1. 69% 2. 3 |
| **Salivary Gland Carcinoma Specific Part** | | |  |
| 9 | In case of close margins after surgical resection of tumors with high aggression do you propose adjuvant radiotherapy? | 1. Yes, in all cases 2. No 3. Yes, in cases of presence of other unfavorable factors | 1. 80% 2. 19% 3. 1% |
| 10 | Please rate the degree of importance of the following factors in your decision making (Very Important – Important – Neutral – Not so Important – Not Important) | 1. Lymphovascular invasion 2. Perineural Invasion 3. T3-T4 stage 4. Site (minor vs major salivary gland) | See table S2 |
| 11 | In case of positive margins after surgical resection (R1 resection) which factors influence your decision between re-resection and radiotherapy? (Multiple choice is allowed) | 1. Site 2. Grade 3. Functional Impairment 4. Time since surgery 5. Nodal involvement 6. Perineural involvement 7. Lymphovascular invasion 8. Other (please specify) | 1. 70% 2. 33% 3. 85% 4. 37% 5. 44% 6. 49% 7. 29% 8. 11% |
| 12 | In case of R0 resection of a T1-T2, cN0 high aggression SCG, what is your treatment approach? | 1. Close follow-up 2. Elective neck dissection 3. Adjuvant lymph-node field radiotherapy in every case 4. Adjuvant lymph-node field radiotherapy in case of need of radiotherapy on primary site (ie. if persence of perineural invastion and/or lymphovascular invasion) 5. Other (please specify) | 1. 37% 2. 14% 3. 6% 4. 38% 5. 5% |
| 13 | In case of R0 resection of a T3-T4, cN0, high aggression SCG, do you propose an adjuvant lymph-node field radiotherapy? | 1. Yes 2. No 3. Depending on primary site, histology, and previous neck dissection 4. Other, please specify | 1. 48% 2. 6% 3. 44% 4. 2% |
| 14 | Is there, in your opinion, a need for contouring guidelines to define the target volume in patients treated with radiotherapy for SGC? | 1. No 2. Yes 3. Not my area of expertise | 1. 2% 2. 75% 3. 23% |
| 15 | In patients treated with adjuvant radiotherapy, when do you prefer particle therapy (proton or carbon ions) over photons? | 1. Based on primary tumor site 2. Based on tumor histology 3. Only in a clinical trial 4. I can’t offer particle therapy due to logistic issues 5. Based on patient's age (less than 65 years old) and performance status (ECOG 0-1) 6. In case of non-resectable macroscopic residual disease at post-operative MRI 7. Never 8. Other (please specify) | 1. 15% 2. 13% 3. 16% 4. 21% 5. 1% 6. 11% 7. 7% 8. 16% |
| 16 | In patients treated with adjuvant radiotherapy, when do you combine radiotherapy with chemotherapy? (multiple choices are allowed) | 1. Based on primary tumor site 2. Based on tumor histology 3. In case of advanced N stage (N2-N3) 4. In case of advanced T stage (T3-T4 5. Based on age (less than 65 years old) 6. Based on performance status (ECOG 0-1) 7. In case of extranodal extension 8. In case of perineural invasion 9. In case of lymphovascula invasion 10. In case of non-resectable macroscopic residual disease at postoperative magnetic resonance imaging 11. Only in a clinical trial 12. Never 13. Other (please specify) | 1. 1% 2. 24% 3. 28% 4. 16% 5. 5% 6. 15% 7. 36% 8. 13% 9. 11% 10. 30% 11. 37% 12. 23% 13. 6% |
| 17 | In case of HER2 Positive SCG (either HER2 3+ on immunochemistry or FISH), when do you offer adjuvant targeted treatment (eg. traztuzumab)? Multiple choices allowed | 1. Based on primary tumor site 2. Based on tumor histology 3. In case of advanced N stage (N2-N3) 4. In case of advanced T stage (T3-T4 5. Based on age (less than 65 years old) 6. Based on performance status (ECOG 0-1) 7. Only in a clinical trial 8. In case of non-resectable macroscopic residual disease at postoperative magnetic resonance imaging 9. Never 10. Other (please specify) | 1. 1% 2. 11% 3. 18% 4. 18% 5. 2% 6. 6% 7. 50% 8. 23% 9. 25% 10. 10% |
| 18 | In case of androgen receptor (AR) positive SGC, when do you offer adjuvant targeted treatment (eg leuprolide and/or bicalutamide)? Multiple choices allowed | 1. Based on primary tumor site 2. Based on tumor histology 3. In case of advanced N stage (N2-N3) 4. In case of advanced T stage (T3-T4 5. Based on age (less than 65 years old) 6. Based on performance status (ECOG 0-1) 7. Only in a clinical trial 8. In case of non-resectable macroscopic residual disease at postoperative magnetic resonance imaging 9. Never 10. Other (please specify) | 1. 0% 2. 13% 3. 15% 4. 13% 5. 4% 6. 7% 7. 51% 8. 28% 9. 22% 10. 9% |
| 19 | Do you refer patients with adenoid cystic carcinoma treated with particle therapy for carbon ion or proton therapy? | 1. Proton 2. Carbon ion 3. I usually do not propose particle therapy for adenoid cystic carcinoma patients | 1. 21% 2. 27% 3. 52% |
| 20 | In case of inoperable (due to extension of disease or comorbidity) locally or locoregional advanced SGC, which treatment do you propose? (multiple choice is allowed) | 1. Radiotherapy with photon therapy 2. Radiotherapy with particle therapy 3. Chemoradiotherapy 4. Radiotherapy + targeted anti-HER2 treatment (if HER2 positive) 5. Radiotherapy + targeted anti-AR treatment (if AR positive) 6. Other (please specify) | 1. 38% 2. 36% 3. 35% 4. 28% 5. 27% 6. 24% |
| 21 | In patients with locoregional relapse treated with surgery, please rate the degree of importance of the following factors in your decision making on adjuvant radiotherapy (Very Important – Important – Neutral – Not so Important – Not Important) | 1. Disease Interval 2. Previous radiotherapy 3. Tumor grade 4. Tumor histology 5. R1 resection 6. R2 resection 7. Presence of extranodal extension 8. Presence of perineural invasion 9. Presence of lymphovascular invasion 10. rT stage 11. rN stage 12. Performance status 13. Age 14. Only in a clinical trial 15. I never propose adjuvant radiotherapy in case of locoregional relapse | See table 3 in the manuscript |
| 22 | When do you propose a molecular characterization of Salivary Gland Carcinoma? | 1. Always at diagnosis 2. At diagnosis in case of high aggression disease 3. At tumor relapse 4. When curative treatment is not feasible, and patient is a candidate for systemic treatment 5. Just to evaluate possible inclusion in a clinical trial 6. Other (please specify) | 1. 20% 2. 13% 3. 19% 4. 41% 5. 4% 6. 4% |
| 23 | Which potentially targetable molecular alterations do you prefer to test? (multiple choices possible) | 1. HER2 in salivary duct carcinoma and salivary gland adenocarcinoma 2. AR in salivary duct carcinoma and salivary gland adenocarcinoma 3. NTRK in acinic cell carcinoma and mammary analogue secretory carcinoma (MASC) 4. Tumor mutational burden (TMB) in all histologies 5. Microsatelite instability (MSI) in all histologies 6. BRAF in all histologies 7. RET in all histologies 8. Whole exome NGS regardless of tumor histology 9. Other (please specify) | 1. 86% 2. 84% 3. 64% 4. 24% 5. 29% 6. 27% 7. 17% 8. 34% 9. 9% |
| 24 | Which factors influence your decision to start systemic treatment rather than to closely follow-up patients? | 1. Tumor histology 2. Disease-free interval between termination of prior therapy and relapse 3. Symptoms 4. Burden of disease 5. Pace of disease 6. Age 7. Gender 8. Presence of liver or bone metastasis 9. Patient preferences 10. Risk of toxicity 11. Availability of clinical trial | See table 4 in the manuscript |
| 25 | What is the percentage of patients with RM SCG with no available targeted treatment to whom you propose systemic chemotherapy | 1. 0 % 2. 1-10 % 3. 10-20% 4. 20-50% 5. > 50% | 1. 1% 2. 23% 3. 21% 4. 29% 5. 26% |
| 26 | In a candidate for systemic treatment with both HER2 positive and AR positive SGC, which systemic treatment do you prefer in the first line? | 1. HER2 targeted treatment (eg. Trastuzumab) 2. AR targeted treatment (eg leuprolide and/or bicalutamide 3. Combination of HER2 and AR targeted treatment 4. HER2 targeted treatment + chemotherapy 5. AR targeted treatment + chemotherapy 6. Chemotherapy | 1. 28% 2. 21% 3. 18% 4. 23% 5. 1% 6. 8% |
| 27 | As this survey emphasizes the existence of many "grey zones" in SGC management, would you be interested in participating in studies to answer some of them? | 1. Yes 2. No | 1. 90% 2. 10% |
| 28 | We aim at investigating some topics of SGC approach, would you indicate what your preferences are? please comment |  |  |
| 29 | In case you are interested, please add your contact email address below | 1. No, not interested 2. Yes |  |

Table S1: complete questionnaire


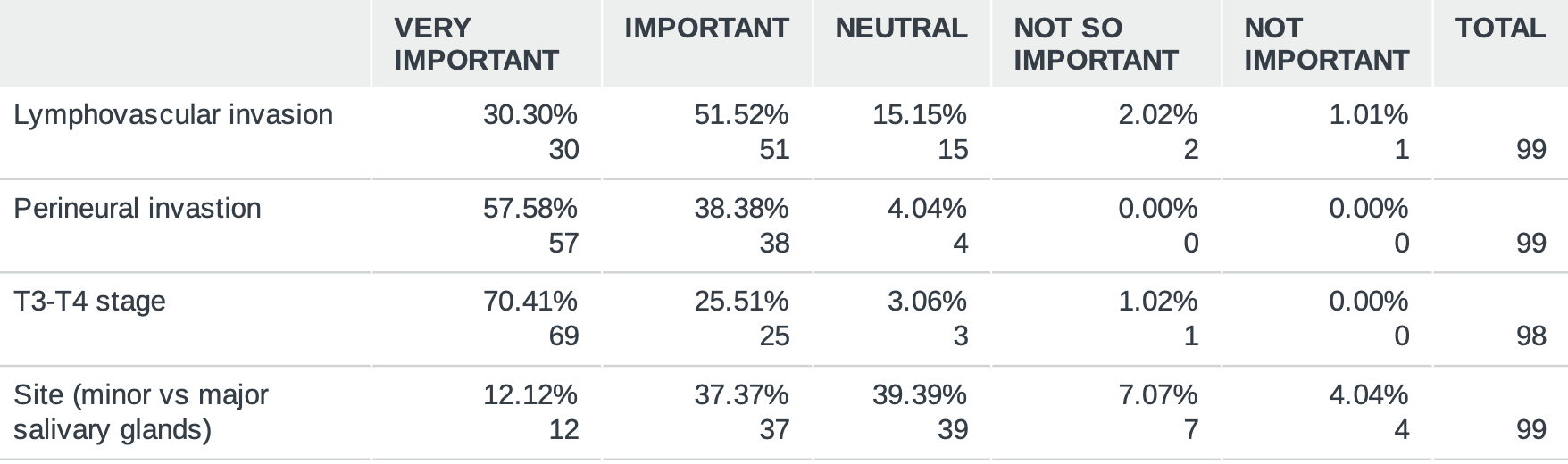


Table S2 Question 10:In case of close margins after surgical resection of tumors with high aggression please rate the degree of importance of the following factors in your decision making to propose adjuvant radiotherapy
